# Salvage after Retroperitoneal Kidney Allograft Torsion

**DOI:** 10.1155/2020/8024598

**Published:** 2020-01-14

**Authors:** Justin M. Greco, David C. Mulligan, Peter S. Yoo

**Affiliations:** Division of Transplantation, Department of Surgery, Yale University School of Medicine, New Haven, CT, USA

## Abstract

Torsion of a transplanted kidney into the retroperitoneal space is a rare occurrence, with only three other reported cases. Failure after kidney transplantation is caused by surgical, immunological, and infective complications. Torsion is a complication that poses a serious risk of ischemic graft failure, and, if suspected, sonographic evaluation helps ascertain the diagnosis. Here, we present the case of a 69-year-old transplant recipient whose routine postoperative ultrasound confirmed vessel patency, however subsequently developed clinical signs of renal allograft compromise. Repeat ultrasound showed signs of vascular compromise and the patient was emergently re-explored. Torsion of the renal allograft about its pedicle was encountered and corrected by manual detorsion and nephropexy to the retroperitoneal wall. Clinicians should recognize pedicle torsion as a potential cause of renal allograft failure and the role of nephropexy in its management.

## 1. Introduction

A total of 19,128 kidney transplants were performed in 2016, and allograft failure is a devastating complication following transplant [1]. Early postoperative causes of allograft loss result from acute rejection, renal vein thrombosis, or renal artery stenosis. Torsion of kidneys transplanted into the retroperitoneal space is a rare complication. Only three other cases have been reported, all within the last ten years [2–4]. These cases represent examples of warm ischemic time in kidney transplants. Torsion is not uncommon after intraperitoneal transplantation, with cases being documented in the setting of simultaneous pancreas and kidney transplants [5–7]. The presumed causative factors are length of the allograft vein, artery, and ureter; location in the peritoneal space; and torque forces of surrounding organs. In contrast, the tight retroperitoneal space should naturally prevent twisting of the renal allograft. Nonetheless, clinical suspicion for this complication and early intervention are critical to salvage a transplanted kidney in any case where vascular compromise is implicated.

## 2. Case Presentation

A 69-year-old woman with autosomal dominant polycystic kidney disease (ADPKD), two years of peritoneal dialysis and two years of hemodialysis, underwent a donation after brain death kidney transplant. ADPKD had affected her mother, maternal grandmother, aunt, and uncle. In addition to renal failure, the patient's medical problems included hypertension, secondary hyperparathyroidism, and arthritis. Her calculated panel of reactive antibodies was 71%. Past surgical history included tonsillectomy, adenoidectomy, appendectomy, and cholecystectomy. The patient reported rare alcohol use and a remote 0.5 pack year smoking history. The patient had a preoperative weight of 57.2 kg and a BMI of 25.89.

**Figure 1:**
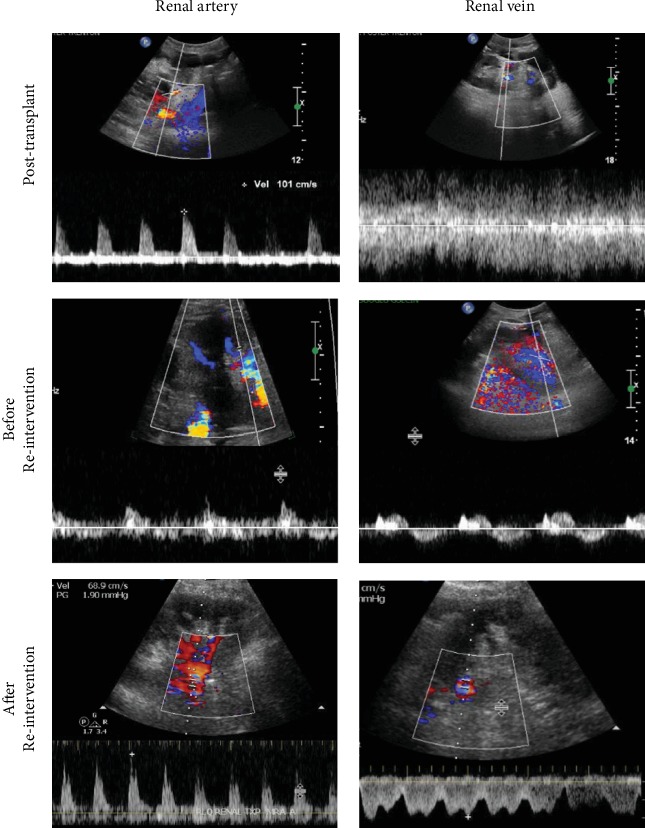
Ultrasound findings of the renal allograft vein and artery.

The kidney was transplanted into the retroperitoneum via a Gibson incision in the right iliac fossa after 13 hours and 52 minutes of cold ischemic time. The graft was from a female donor, right-sided, and 11 cm in length. Several renal cysts in the native kidney were drained to make space for the transplant. The renal allograft pedicle consisted of a single artery, vein, and ureter; there was not an aortic patch. The donor renal artery and vein were anastomosed to the recipient external iliac vessels, in an end-to-side fashion. A J-stent was placed across the uretero-vesical anastomosis. The initial intraoperative urine output was low but, substantially increased to 1270 mL during the first 4 hours. Preoperative serum creatinine declined from 6.51 mg/dL to 3.20 mg/dL after completion of the procedure, and the blood urea nitrogen (BUN) also decreased from 47 mg/dL to 17 mg/dL. In keeping with our institution's standard of practice, an immediate allograft ultrasound was done in the post-anesthesia recovery unit which demonstrated normal flow dynamics and elevated resistive indices, with no hydronephrosis or perinephric collection (Figure 1).

Over the next 18 hours, the urine output gradually dropped to 321 mL, at a rate less than 0.5 mL/kg/hr, which did not respond to an intravenous fluid challenge. This prompted a repeat ultrasound of the renal allograft. Tardus parvus waveforms and nonvisualization of the renal vein were observed, which was concerning for arterial stenosis, arterial thrombosis, or venous thrombosis. The patient was emergently dialyzed and taken to the operating room 30 hours after the initial procedure. On re-exploration, the kidney allograft was found to be rotated 180 degrees clockwise with near total occlusion of the renal artery, vein, and ureter.

Detorsion of the kidney resulted in restored perfusion, palpable pulse, and good Doppler signals in both the artery and vein. Normal turgor and pink color returned. The allograft was then secured by a nephropexy to prevent future torsion using a superior pole silk suture and application of Arista potato starch, to induce scarring. During the next four postoperative hours, the urine output improved to 881 mL and the Doppler ultrasound showed good renal vein flow and absence of tardus parvus waveforms (Figure 1). In parallel, the creatinine decreased from 3.68 mg/dL to 2.60 mg/dL and BUN dropped to 25 mg/dL from 36 mg/dL.

The patient was discharged on postoperative day 10. However, she experienced further complications leading to multiple readmissions. These included ureteral stent migration and replacement, percutaneous drainage of a periallograft hematoma on two occasions, deep vein thrombosis requiring placement of an inferior vena cava filter, and urosepsis. The patient's last discharge was 56 days after the transplant, and she has remained well since.

## 3. Discussion

Only three other cases of retroperitoneal renal allograft torsion have been reported in the literature. In 2009, Ozmen et al. suspected malposition of the kidney when urine output dropped significantly on postoperative day 5, leading to the identification of allograft torsion on ultrasound (Table 1) [2]. Winter et al. described a case of torsion they believe resulted from the patient's increased BMI and error in surgical technique [4]. In 2014, Sosin et al. reported laxity of the abdominal wall and accompanying potential large retroperitoneal space as the primary factors leading to rotation of the graft [3]. We believe that our case represents yet another situation whereby an enlarged native polycystic kidney exerted pressure and caused rotation of the renal allograft about its pedicle. Given this patient's BMI and small frame, torsion was further facilitated with postoperative patient movement and ambulation. The time interval to intervention in all these cases ranged from as little as 4 hours to 5 days, but in each instance, the kidney transplant was salvaged. Decreased urine output followed by sonographic postoperative evaluation demonstrating compromised arterial and venous flow prompted operative exploration. Altogether, these four cases should serve to raise awareness of this serious but reversible complication. Furthermore, transplant surgeons should consider nephropexy as a means of reducing the risk of renal allograft torsion in retroperitoneal transplants.

## Figures and Tables

**Table 1:** Allograft torsion after retroperitoneal renal transplants.

Date	Authors	Patient	Transplant indication	Ischemic time	Ultrasound findings	Time to reintervention	Proposed mechanism	Post-op course
2009	Ozmen, et al. 2013	44 yo M	Sepsis and renal failure—received kidney from a living related donor	Not reported, living donor transplant	US showed rotation of renal hilum and Doppler with stenotic flow	POD 5	Malposition of graft kidney	Recovered well
2013	Winter et al.	48 yo M	Hypertension and diabetes mellitus—received a deceased donor's kidney	8 hours and 46 minutes of cold ischemia time	US with absent main renal vein flow and reversed diastolic arterial flow	4 hours	Raised body habitus and/or error in surgical technique	Return of adequate UOP after 1 week, discharged at POD 30
2014	Sosin et al.	42 yo M	Glomerulonephritis—received DCDK	4 hours of cold ischemia time	US at 11 hours post-op: tardus parvus and decreased resistive index	11 hours	Large potential space in iliac fossa, lax abdominal wall	Oliguric, required dialysis, required antithymocyte therapy for acute rejection on POD6, recovered kidney function, and discharged POD8
2018	Present case	69 yo F	ADPKD—received DBDK	12 hours and 52 minutes of cold ischemia time	US immediately post-transplant and 18 hours post-transplant	30 hours	Pressure form native polycystic kidney	As described: ureteral stent migration, hematoma, DVT, urosepsis

Abbreviations: YO: year-old; M: male; F: female; ESRD: End-Stage Renal Disease; POD: postoperative day; DCDK: Donor after Cardiac Death Kidney; DBDK: Donor after Brain Death Kidney; UOP: urine output; DVT: deep vein thrombosis; US: ultrasound.

## Abbreviations

ADPKD: Autosomal dominant polycystic kidney disease
BUN: Blood urea nitrogen.
